# High Performance of Covalently Grafting onto Collagen in The Presence of Graphene Oxide

**DOI:** 10.3390/nano8090703

**Published:** 2018-09-09

**Authors:** Zahra Bazrafshan, George K. Stylios

**Affiliations:** Research Institute for Flexible Materials, Heriot Watt University, Galashiels TD1 3HF, UK; g.stylios@hw.ac.uk

**Keywords:** biocomposite, collagen, graphene, physical properties, mechanical testing, casting, electrospinning

## Abstract

A collagen-based copolymer, ASC-g-Poly(methyl methacrylate-co-Ethyl Acrylate), was synthesized in the presence of Graphene Oxide (GO) via an in-situ polymerization. The presence of GO that increased the accessible surface area for initiated collagen chains allowed for an accelerated polymerization with highly improved grafting performance and efficiency. This was conducted from two polymerization systems with varied comonomer feed ratios, in which two distinguished GO loadings were used. The processability of the achieved nanocomposite was then evaluated through casting and electrospinning processing methods. Fourier Transform Infrared Spectroscopy (FT-IR), UV-Vis spectroscopy, Differential Scanning Calorimeter (DSC), Thermogravimetric analysis (TGA), Scanning Electron Microscope (SEM), Transmission electron microscopy (TEM), and tensile analysis were conducted to characterize the GO-ASC-g-P(MMA-co-EA). The nanocomposite films showed a unique morphology, multilayer nanostructure of the grafted GO monolayers that deposited simultaneously one on top of another. The morphology of the electrospun fibers was affected by the addition of GO loadings in which the increase in fiber diameter was observed while the surface of the nanofibers was decorated by the GO nanolayers. To modify the collagen, this research highlights the importance of introducing functional groups of GO and the substitution of GO loadings as an active nanostructure filler to highly monomer feed ratios improving the physiochemical properties of collagen. This easy-to-apply approach is suggested for applications intending the mechanical properties and deterred degradation of Collagen-based materials.

## 1. Introduction

Nanofillers due to owning a large surface-to-volume ratio have developed as smart candidates as fillers [1,2,3]. Their large specific interfacial area allows for them to theoretically have higher interactions with polymer matrix [3,4]. The relatively newest class of two-dimensional carbon based nanofiller is graphene and its derivatives that have found a variety of applications, to promote the polymer matrix [5,6,7]. Nevertheless, due to undesired interactions within the polymer matrix, its processability is still problematic [1,2]. The nanofiller dispersion in the polymer matrix is all the more emphasized aspect of processing techniques wherein the nanofillers are likely to agglomerate [1,8,9]. 

Several advantages that graphene can devote to the host (co)polymer, include reinforced mechanical properties, such as modulus and tensile strength, as well as dimensional stability and increased heat resistance [10]. Among them, graphene as a mechanical reinforcement can be the most beneficial approach in bio-based materials that are typically suffering from poor mechanical properties and stability in high humidity conditions [2,4,6,10]. The extent of enhancement induced from Graphene is directly related to its exfoliation and dispersion thereafter. 

A widely used biomaterial is collagen and its derivatives [1,6,11]. This bio-based polymer is purified by chemical degradation, which leads to the triple helix into the random-coil structure of collagen [1,12,13,14,15,16]. Collagen as a biocompatible and biodegradable biopolymer justifies several usages for a variety of applications in medical fields [13,14,16]. The purified collagen cannot be used solely due to its drawbacks, such as poor mechanical properties and increased hydrophilic behavior that can accelerate the degradation rate. But, due to its excellent properties [17,18], it has been processed with a series of compatible materials to enhance its properties [19,20,21,22,23,24,25,26]. 

To reduce the superhydrophilicity of the purified collagen chains, a variety of monomers can be randomly branching over the surface of collagen chains. Thereby, the modified collagen can benefit from the newly achieved structure while receiving some of the physiochemical properties of grown branches, such as thermal and mechanical. The main drawback of this conventional methodology can be its low performance by contrast with its by-products wherein the desired conversion is randomly achieved onto the host polymer. Several attempts have been devoted to optimizing the synthesis parameters to increase the grafting performance (grafting yield) onto collagen. Among them, the increased monomer feed ratio plays a key role in the grafting performance while it can be less desired in large-scale production due to highly formed by-products. However, to the best of our knowledge so far, the effect of the presence of graphene-based nanofillers in the performance of in-situ polymerization onto collagen has not gained attention. 

Graphene oxide (GO) is the widely employed form of the modified graphene [6,10,27]. GO has a layered structure with numerous oxygen functionalities (epoxide, hydroxyl, carboxyl, and etc.) on the basic planes and edges [11,27,28] and Collagen as a polyelectrolyte can be identified by the amine and carboxyl groups. Thereafter, covalent bonds, Vander Waals forces, such as hydrogen bonding, electrostatic interaction, or π–π stacking between collagen chains, macromonomers, and GO are expected to elevate the grafting performance and the stability of the nanocomposites.

Hence, the novelty of this work is to benefit the presence of Graphene oxide to enhance the output of collagen-based nanocomposites during the synthesis and process in quality and quantity. The importance of this work is due to (i) using water as medium of polymerization in which GO can be highly dispersed, (ii) increasing the efficiency and the yield of the polymerization when GO can grafted to either the collagen chain as host polymer and introduced monomers, (iii) facilitating the process of the obtained nanocomposite due to the elimination of challenges regarding the GO dispersion and the GO re-agglomeration while processing, and (iv) GO as a reinforcing agent with highly active functionalities can simply improve the functionality of the collagen-based nanocomposites when the mechanical properties and deterred degradation rate of collagen are tailored in use. The processability of the collagen-based nanocomposite was investigated while using a casting and electrospinning methodology. The nano-layered composite structure was achieved by casting methodology that can be potentially an attractive candidate for applications in nanomechanical systems, and hydrophilic transparent and paper-like collagen-based nanocomposites. 

Furthermore, to mimic the structure of the collagen fibrils in native tissues, the collagen-based composite was processed by using electrospinning. A slightly increasing in fiber diameter was observed by increasing the GO content that can be due to varied cause and effects that can be further optimized based on the preferences. Therefore, in this study, a set of six samples synthesized by in situ polymerization onto Acid soluble collagen (ASC) was examined to consider the influence of GO loadings on nanocomposite performance. 

## 2. Experimental Section

### 2.1. Materials

Collagen from cow skin was provided by Devro Plc., Moodiesburn, UK. Methyl methacrylate (MMA, 99%, Alfa Assar, Heysham, UK) and Ethyl Acrylate (EA, 99%, Alfa Assar, Heysham, UK) were used as monomers and were passed through a column of 5% sodium hydroxide aqueous solution to remove an inhibitor existing in the monomers. Benzoyl peroxide (BPO,97%, Alfa Aesar, Heysham, UK) was used as initiator and recrystallized in Acetone before applying. Graphite flake (99%, Alfa Aesar, Heysham, UK), Acetic acid (AA, 99.7%, Alfa Aesar, Heysham, UK), Formic acid (FA, 99%, Alfa Aesar, Heysham, UK), sulfuric acid (H2SO4, 95–98%, Alfa Aesar, Heysham, UK), Phosphoric acid (H3PO4, 85%, Alfa Aesar, Heysham, UK), potassium permanganate (KMnO4, 99%, Alfa Aesar, Heysham, UK), and methanol (MeOH, 99.9%, Alfa Assar, Heysham, UK) were applied as received. 

### 2.2. Synthesis of GO-ASC-g-P (MMA-co-EA) 

GO was synthesized from natural graphite flakes (1.5 g) while using modified Hummer’s method, purified, and dried [27,29]. More specifically, a 9:1 mixture of concentrated H2SO4/H3PO4, (180:20 mL) was added to a mixture of graphite flakes and KMnO4 (9.0 g). The reaction was then heated to 50 °C and stirred for 12 h. The reaction was then cooled to room temperature by pouring onto cubic ice (200 mL) mixed with H2O2 (1.5 mL). The mixture was filtered through a Polytetrafluoroethylene (PTFE) membrane (0.2 µm pore size). The remaining solid material was then washed in sequences with 100 mL of water, 100 mL of HCl (36%), and 100 mL of ethanol; for each wash, being filtered through the polyester membrane. The remained material after this extent was coagulated with 100 mL of ether and was filtered again. The solid remaining on the filter was dried in a vacuum oven at room temperature. 

GO-ASC-g-P(MMA-co-EA) nanocomposite was synthesized by in situ polymerization technique applying benzoyl peroxide as an initiator, a GO aqueous suspension, an ASC solution, and comonomer with desired ratios at 80 °C for 30 min [24]. In a typical preparation, GO powder was dispersed in distilled water and stirred for 1 h using a magnetic stirrer followed by a 15 min sonication to achieve a fully exfoliated GO suspension (13 mg mL^−1^) and then diluted five times in distilled water. The ASC solution was prepared using collagen in diluted AA in distilled water to reach a pH of 2.5 ± 0.5. The mixture was incubated for 5 h at 45 °C in a 250-mL triple necked round bottom flask and a stirrer bar was added. This step was terminated by the suddenly increased temperature of 80 °C, the threshold of achieving ASC in water, as seen as a homogenous solution. N_2_ gas was applied through the solution while stirring. The GO suspension was added to the 150 mL ASC (36 mg mL^−1^) solution within three times in 15 min. After 1 h, dissolved BPO in 2 mL Acetone as the initiator, was added gently to the reaction vessel within 10 min. The mixture of distilled MMA and EA in the rates mentioned in Table 1, were then introduced via a syringe in 30 min. The temperature and reaction time were fixed at 80 °C and 60 min after adding the initiator and the monomers. The stirrer speed was fixed at 2400 rpm during the reaction. Precipitation of the graft copolymer occurred after 10 min of reaction time. The reaction mixture was then added to an excess amount of cool methanol for complete precipitation. As with any free radical copolymerization reaction, the formation of the attendant polymer of P(MMA-co-EA) always arises along with that of the desired nanocomposite (GO-ASC-g-P(MMA-co-EA), owing to reactivity ratio effects or the segregation of macromonomer from main and side chains with the presence of GO. An extraction step was needed to remove ungrafted ASC and GO, unreacted (MMA-co-EA) macromonomer and P(MMA-co-EA) from the desired copolymer product. A simple isolation method of selective solvent extraction based upon the difference in the solubility was employed. Therefore, the resulted product was extracted by repeated washings with boiling water followed by acetone at room temperature to remove by-products using a sintered glass filter under reduced pressure. All the samples were dried in a vacuum oven at room temperature until a constant weight was achieved.

The grafting parameters, i.e., grafting-percentage (*GP*, %) and grafting-efficiency (*GE*, %), were used to characterize the identified copolymer. *GP* shows the increase in weight of original ASC subjected to grafting with comonomers and was calculated using the equation given below:

(1) $$\;GP\left(\%\right)=\frac{W1-W0}{W0}*100\;$$

where *W*0 and *W*1 are the weight of the original ASC and the ASC graft nanocomposite, respectively.

Grafting efficiency (*GE*) indicates the fraction of comonomer grafted onto ASC among the amount of MMA-co-EA converted to graft copolymer plus P(MMA-co-EA) that was calculated using the following equation:

(2) $$\;GE\;\left(\%\right)=\frac{W1-W0}{\left(W1-W0\right)+W2}*100\;$$

where *W*0, *W*1, and *W*2 are the weights of the original ASC, the collagen graft nanocomposite, and P(MMA-co-EA), respectively. The weight of P(MMA-co-EA) (*W*2) can be calculated by subtracting the weight of grafted polymer plus the amount of ungrafted copolymer that was collected from the extraction. Due to the negligible weight of GO when compared with the weight of the host polymer and the monomers, the weight of GO was not counted in the calculations.

The samples were labeled as S1-8, S1-16, S2-8, and S2-16. The collagen graft copolymers without GO content, (ASC-g-p(MMA-co-EA), were then used as the reference and named S1-0 and S2-0. 

### 2.3. Preparation of Films and Nanofibers 

A 10 % (*w*/*v*) solution of each sample was prepared when a mixture of Acetic Acid and Acetone (4:1) was used as a selective solvent. Films were obtained from 10 mL of each solution on the bottom of Petri dishes (6 cm in diameter) after solvent evaporation at room temperature. Then, the film samples were labeled as F1-8, F1-16, F2-8, and F2-16. The thin films of collagen graft copolymers without GO content, (ASC-g-p(MMA-co-EA), were used as the reference and named F1-0 and F2-0. For fiber formation, A Spraybase electrospinning apparatus (Spraybase, Dublin, Ireland) was used consisting of a high voltage power supply, a syringe pump, a syringe and tubing, a 20-gauge needle and a grounded rotating collector (surface length of 25 cm, diameter of 9 cm) at a speed of about 1.7 m/s. The solution was feed, through a Teflon tube, to the needle that was placed in a syringe pump. All the above-mentioned samples were electrospun into nanofibers by using the following electrospinning conditions: applied voltage (10 kV), needle to collector distance (TDS, 12 cm), and solution flow rate (0.5 mL/min, room temperature and relative humidity (*RH*, 30–35%). Electrospun nanofibers were labeled according to GO contents, as following; M1-8, M1-16, M2-8, and M2-16. 

### 2.4. Characterization

The structure of the nanocomposite films was considered by Fourier Transform Infrared Spectroscopy (FT-IR, Thermo Nicolet Avatar 370 DTGS, Thermo Electron, Madison, WI, USA) at room temperature. UV-Vis spectra were recorded by UV-VIS Perkin Elmer Lambda 35 Spectrometer (PerkinElmer, Singapore). Thermal analysis of the films was performed by Differential Scanning Calorimeter (DSC, Mettler DSC 12E, Mettler-Toledo Ltd., Leicester, UK). Temperature ranges from 23 °C up to 280 °C and vice versa for cooling scans with a heating/cooling rate of 10 °C min^−1^ in a nitrogen atmosphere was performed. A three minute-time remaining at 300 °C was applied to erase the history of the thermal behavior of the samples (7 mg) for evaluating changes during the cooling scans. Endotherms were represented with upward curves in the scans. Thermogravimetric analysis (TGA) was carried out by using a thermogravimetric analyzer (Mettler TC 10A/TC 15 Instrument, Mettler, Zürich, Switzerland). The film samples were heated from 35 °C to 600 °C at 10 °C /min monitoring the sample weight variant. Morphological investigation of the composite samples was performed at 3kV while using a Hitachi 3400 series Scanning Electron Microscope (SEM, Hitachi Ltd., Tokyo, Japan) and a Transmission electron microscopy (TEM, FEI Tecnai F20 S/TEM, FEI Company, Hillsboro, OR, USA). The films and the electrospun nanofibers were coated with a gold thin film before SEM imaging to ensure higher conductivity. TEM was used to represent in-depth details of the structure and the arrangement of the nanofillers within the fibers. The mean fiber diameter and the uniformity of the fibers (standard deviation value) were determined statistically by using ImageJ software from SEM micrographs through the measurement of about 150 fibers and the results were given as the average diameter ± standard deviation that shows the uniformity of the fibers. Mechanical characterization was studied on strip-shaped (50 mm × 10 mm, a thickness of around 25 µ determined by micrometer measuring tool. Stress-strain curves were recorded on relaxed samples over a night (temperature: 22 °C, *RH* = 62%) while using an INSTRON Testing Machine (3345 series, Instron, Canton, CA, USA) with a load cell of 500 N capacity under standard atmospheric conditions for testing. The crosshead speed was set at 5 mm/min for both cases of the films and the electrospun fibers. The Young’s modulus (*E*), tenacity, stress at break, and strain at break of the strips were statistically determined in which five specimens were evaluated for each sample. The results were calculated as the average value and the associated standard deviation. Swelling behavior (hydration ratio) of the fiber samples (about 0.05 g) was immersed in 20 mL Phosphate-buffered saline (PBS, pH 7.4) for 12 h at room temperature and the wet weight (Mt) was measured after blotting with a filter paper. The hydration ratio of the nanocomposites was calculated, according to the following equation:

(3) $$\;Hydration\;ratio\left(\%\right)=\frac{Mt-M0}{M0}*100\;$$

where *M*0 is the weight of the sample before immersion in PBS.

The measurements were performed on five replicate samples. The degradation was calculated by dividing the lost weight of the samples by the original weight of the samples. The degradation evaluations were achieved, as defined by Zhu et al. [30].

All the statistical values were reported as means and standard deviation. Statistical analysis was performed using a one-way analysis of variance (ANOVA) in Excel 2016 with significance being set at *p* < 0.05. 

## 3. Results and Discussion

**Figure 1:**
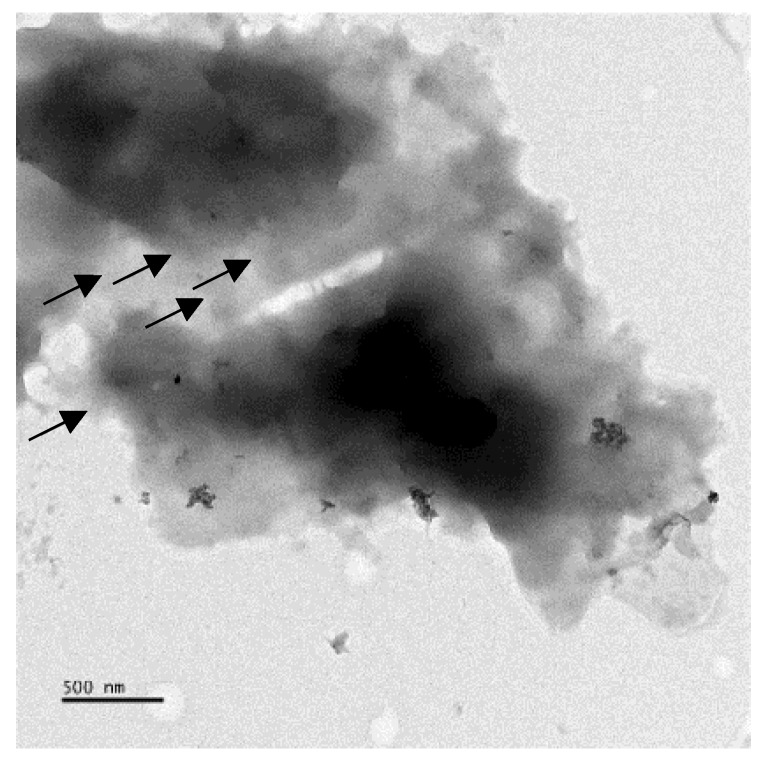
TEM image of few-layer Graphene oxide that (Black arrows) stacked on top of one another. The scale bar shown on the bottom bar is 500 nm.

At the first step, the modified Hummers method was employed to develop the GO by the oxidative exfoliation of graphite flakes. The TEM image of few layers of GO was shown in Figure 1. ASC-GO complex was then successfully achieved. According to literature, GO provides good synergetic effect when mixed with collagen due to having a high concentration of oxygen functionalities [5,11]. For an instant, a GO/collagen-based hydrogel was recently synthesized by melt-coupling reactions of an aqueous GO suspension and a gelatin-water solution at 95 °C. They claimed that the amines can be conjugated onto GO wherein reducing them into Reduced GO [11]. This reaction can be considered by two main routes; the amidation reaction of carboxylic acid groups on the GO edges and also ring-opening amination of epoxy on GO surface [5,11]. Hence, both reactions are likely to occur for the amino groups of collagen chains. Furthermore, the hydrogen bonding happening between amine and hydroxyl on the GO can also be proposed [1,6,10,11,31,32]. Although the detail of chemical reactions is not clear due to the complexity of GO structure, it is hypothesized that the main interactions between ASC and GO during the synthesis and the process could be as noticed above. 

In the next step, the synthesis strategy included grafting MMA-co-EA onto ASC-GO conjugated complex by in situ polymerization. Therefore, due to the physiochemical interactions between ASC and GO, ASC is likely to be coupled on GO, whilst P(MMA-co-EA) chains were grown and covalently bonded onto ASC backbone via free radical polymerization at a temperature below the denaturation process temperature of ASC (80–110 °C). Therefore, we evaluated this hypothesis to preserve the higher fraction of ASC in the desired product of in situ polymerization benefiting from the presence of GO and the monomers as coupling reagents. 

As shown in Table 1, a significant increase was achieved in *GP (%)* by increasing the GO contents, that confirms the notable increase in weight of ASC subjected to grafting with GO and P(MMA-co-EA). Another significant increase that achieved in GE indicates the higher fraction of MMA-co-EA that converted into the tailored graft copolymer. This enhancement is obviously due to the presence of GO that provides a feasibility for reactants to bond to the variety of its functional group on the surface and the edges. Therefore, the produced composite structurally modified to a branched collagen-based macromolecular that grafted with GO, even though there is a high probability for macromonomers to be grafted onto GO individually as well. This covalent bonding of comonomer on the GO surface is unavoidable, even though can be counted as a good opportunity for the composite to benefit from the interfacial repulsions preventing for reagglomerating of GO in the solvent while processing. However, as above mentioned, benefitting from a higher ASC graft copolymer (*GP*) through the presence of GO contents is the main objective of this work. 

**Figure 2:**
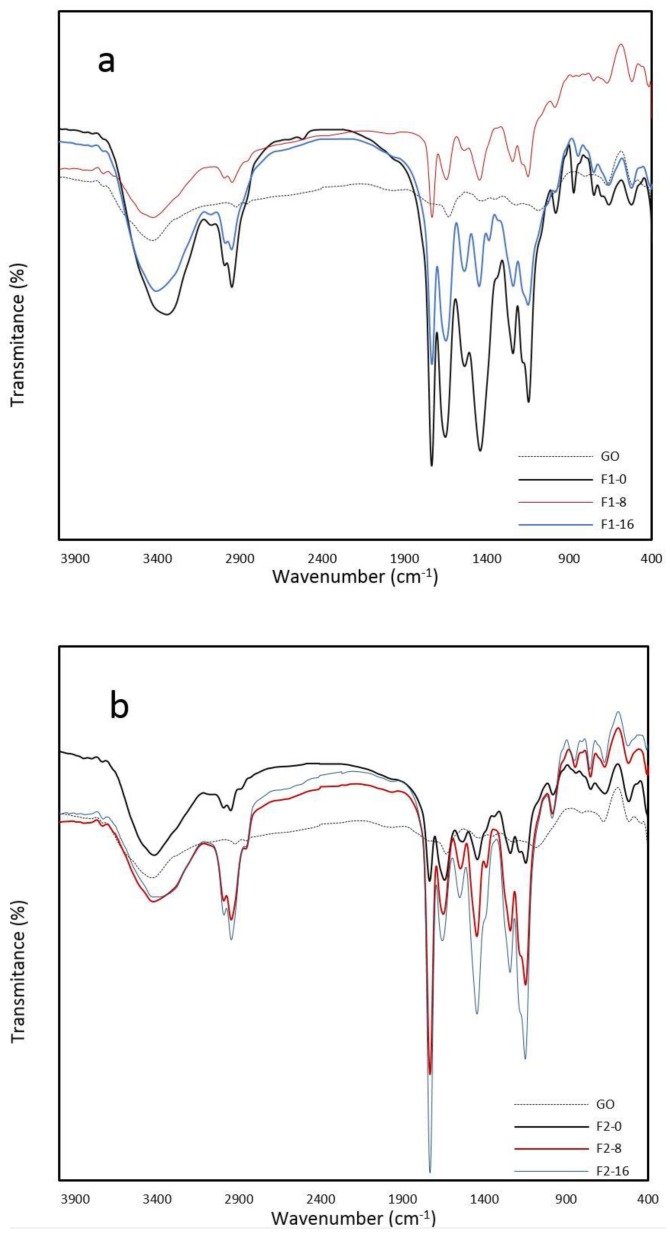
Fourier Transform Infrared Spectroscopy (FT-IR) Transmittance vs. frequency for ASC-g-P(MMA-co-EA) with/without graphene oxide (GO) contents; to clarify, curves associated with comonomer feed ratios were demonstrated, as following: (**a**) composite films of lower comonomer feed ratio and (**b**) composite films of higher comonomer feed ratio.

To investigate the structure of ASC-g-P(MMA-co-EA) with the presence of GO, the FT-IR spectra of the film samples were studied. For more clarity in the variation of the FT-IR peaks based on GO contents and monomer feed ratios, the spectra were shown in two graphs. The FT-IR spectrum of GO, Figure 2a,b, shows the presence of different oxygen functionalities: carbonyl groups (C=O, 1735 cm^−1^, 1640 cm^−1^), (broad stretching vibration C=C in rings, 1550–1710 cm^−1^), alkoxy groups (C–O, 1070 cm^−1^, 1370 cm^−1^), epoxy groups (C–O–C, 1225 cm^−1^), (O–H stretching bond at 3539 cm^−1^), and also C–H stretching symmetric bands at 2852–2906 cm^−1^. 

F1-0 and F2-0 were characterized for comparison wherein synthesized without GO, and its main characteristic groups are identified in Figure 2a,b, respectively. In the case of F1-0, the absorption bands at 2991–2948, 1733, 1536–1644, 1441, 1148, 1242, and 3100–3500 cm^−1^ are assigned to the asymmetric stretching vibration of C-H, strong stretching vibration (C=O), asymmetric vibration of NH2 (amide I & II), CH3 and CH2 bending deformation, stretching vibration of the C–O bond in the C–O–C moiety, stretching vibrations (C–N), and amide A broad stretching vibration (N-H/OH), respectively. In the case of the FT-IR spectrum of F2-0, the profile is almost the same as F1-0 in different intensities without showing any other peaks.

Upon in situ polymerization with the presence of GO and P(MMA-co-EA), the intensity of oxygen functionalities in the corresponding FT-IR peaks were weakened in lower GO contents in contrast to F1-16 and F2-16, wherein the intensity of the FT-IR peak of the C=O at 1730 cm^−1^ was increased with increase in the GO contents due to contribution of C=O groups from P(MMA-co-EA). The amide I vibration and amide II bending vibration from ASC-g-p(MMA-co-EA) dominate in the studied nanocomposites with GO contents, overshadowing the C=C vibrations from GO at 1640 cm^−1^. 

The feature of the Amide I and II was slightly shifted to a higher frequency due to hydrogen bonding between GO and ASC-g-p(MMA-co-EA) in samples with higher P(MMA-co-EA) contents (F2-8 and F2-16), wherein a new peak of CH3 deformation bending vibration was observed nearly at 1387. The reduced oxygen functionalities of GO and similarities of the main features to ASC-g-p(MMA-co-EA) indicated the dominance of polymer segment of the nanocomposite and also the presence of newly formed bonds between ASC-g-p(MMA-co-EA) and GO. Two new absorbance peaks can be observed in all samples: asymmetric vibration peak at 978 cm^−1^ referring to α to nitrogen linkage (N–C) and asymmetric stretching at 1158 cm^−1^ corresponding to (CO–O–C) that can refer to the grafting points on the main backbone of ASC.

**Figure 3:**
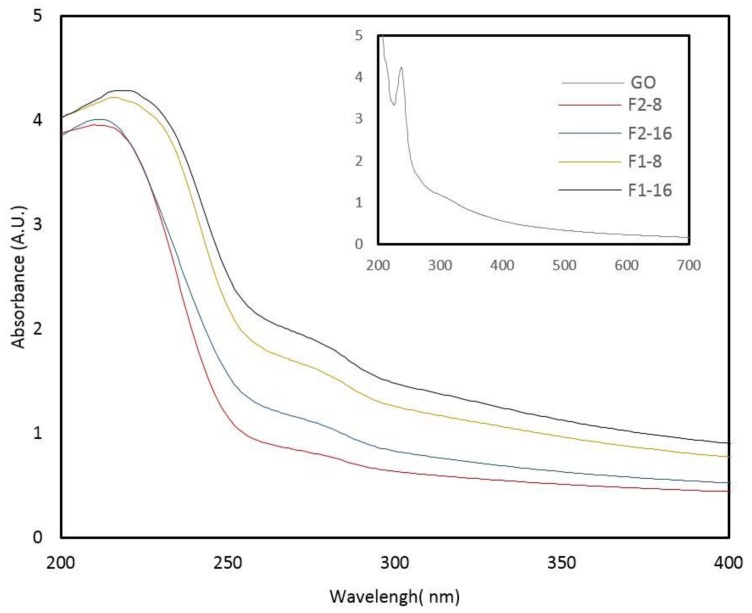
UV-Vis spectra of composite films from GO-ASC-g-P(MMA-co-EA) suggesting π–π stacking of the components with increasing GO contents.

To identify the GO content, UV-Vis spectroscopic studies were performed, Figure 3. It is inferred that the presence of GO contents is identified through the optical absorption of GO dominated by the π–π stacking at about the more intense peak of 230 nm [33,34]. The π–π stacking peak on film samples with GO contents was recorded with a blue shift when compared with GO at 212, 213, 216, and 220 nm for F2-16, F2-8, F1-8, and F1-16, respectively. This can be due to linking units of GO, such as C–O and C=O, and C=C bonds that altered during in situ polymerization. However, a red shift was observed within the film samples by increasing the GO contents. This can be attributed to owning a higher intensity of C=C and oxygen-containing bands in samples with higher GO contents. This statement can be confirmed by the intensity of FT-IR peaks of F1-16 and F2-16, which can be due to the non-uniformity of the GO dispersion in the high loadings that possibly happened during in situ polymerization.

**Figure 4:**
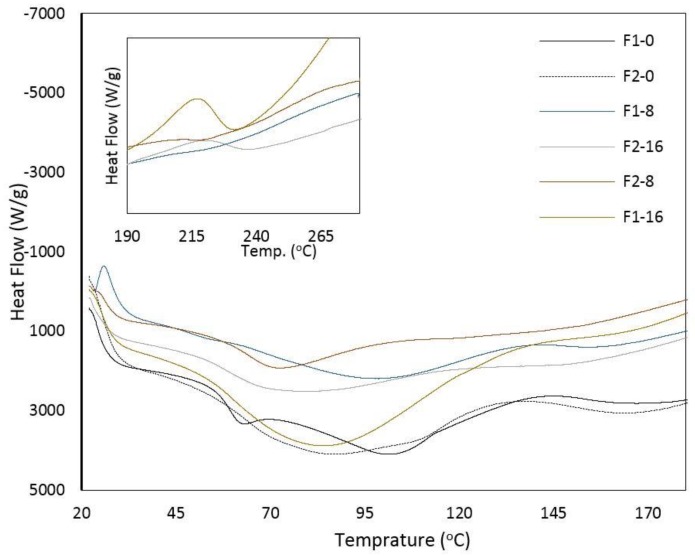
Heat flow vs. temperature of composite films from ASC-g-P(MMA-co-EA) with/without GO contents, in Differential Scanning Calorimeter (DSC) heating phase with the rate of 10 °C min^−1^ in an aluminum pan. To clarify, curves associated with the decomposition transition of GO contents were demonstrated in the same graph happening within the temperature ranges between 190–280 °C) in samples with high GO contents.

The effect of GO content with different loadings on the thermal behavior of GO-ASC-g-P(MMA-co-EA) nanocomposites is depicted in Figure 4. It is known that ASC shows two endothermic events in thermal analysis, referring to melting temperature (Tm) and the denaturation process temperature (Td) [35,36,37]. The first endothermic peak referring to Tm occurs at a temperature between 35–50 °C, depending on the structural hierarchy of peptide chains. The second event, Td, appears at a range of 80–110 °C.

In ASC-g-P(MMA-co-EA), a clear melting peak of ASC was observed at 61.64 °C (F1-0), while this peak could not be clearly observed by increasing the P(MMA-co-EA) segment (F2-0). This can be due to heat transfer carried out by a higher density of P(MMA-co-EA) as the side chain on ASC. On the other hand, one wide endothermic region was observed in F2-0 that can be counted as a complex peak for Tm and Td of ASC at 86.64 °C. In this case, although the Tm of ASC was postponed to a higher temperature by increased density of side chains, the enthalpy changes of this thermal transition (∆H) is approximately the same value of F1-0, 1703 J·g^−1^, and 1523 J·g^−1^, respectively. Also, an obvious melting region at about 160 ± 0.5 °C was recorded with regards to the melting temperature of side chains for both F1-0 and F2-0 without GO content. 

In the samples of GO-ASC-g-P(MMA-co-EA), the above-mentioned complex peaks were observed at a mean value of 87.31 ± 7.34 °C; wherein, the highest temperature (99.28 °C) is associated to F1-8 possessing a less side branch density and less GO contents, and the lowest temperature (74.27 °C) is for F2-8 owning a higher branch density and less GO contents. Therefore, increasing the GO contents has an insignificant effect on Tm and Td of ASC. 

However, unlike the samples without GO, the endothermic transition correlated to side branch melting with the presence of GO were recorded in a lower diversity (standard deviation) at about mean value of 150 ± 5.66 °C. Interestingly, all the samples with GO contents found a decrease in the melting temperature of side chains. This can be due to a reduced average length of side chains when the comonomers grafted onto the ASC chain because of enhanced grafting performance and efficiency. Additionally, ∆H was decreased significantly in endothermic transition events by the GO contents. These results suggest that the thermal stability of ASC based nanocomposite can enhance with the presence of GO in which the increasing temperature can induce exothermic transitions due to reformed carbon-oxygen bonds on the surface and edges of GO.

In the heating phase of the samples with GO contents, an irreversible exothermic event was also observed at 233.13 °C and 221.17 °C that is attributed to the reduction (defunctionalization) of graphene oxide in samples owning higher GO contents (F1-16 and F2-16) (Figure 4). This feature was not observed in DSC profiles that were recorded in the cooling phase (not shown). Defunctionalization, which is ascribed to decomposition of the labile oxygen functionalities, such as –OH, –COOH, and –C=O, can be referred to the density of free functionalities on the surface and edges of GO that probably have not been bonded during in situ polymerization and casting afterward. In the case of F1-8 and F2-8 with less GO contents, it is likely that the formation of covalent bonds within the polymeric matrix renders the oxygen functionalities and this can mark F1-8 and F2-8 less amenable and more stable samples by increasing temperature as compared to other samples.

**Figure 5:**
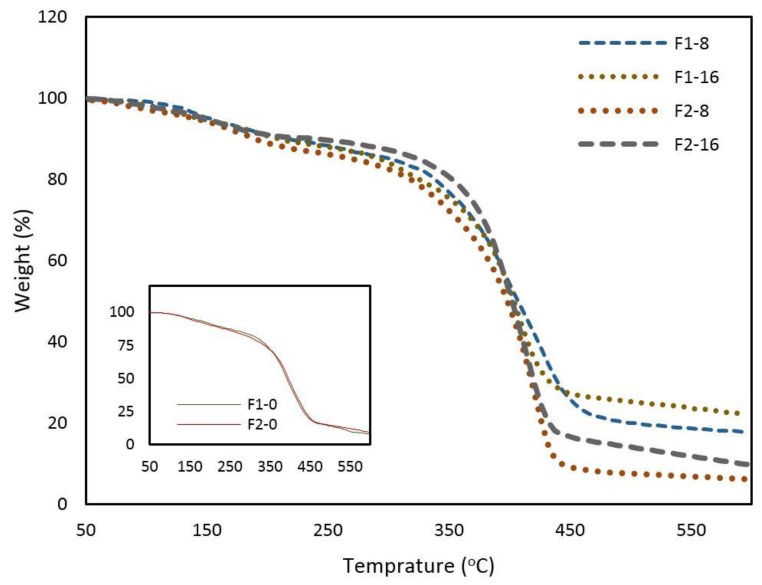
Thermogravimetric analysis (TGA) graph of weight change as a function of temperature for composite films from ASC-g-P(MMA-co-EA) with GO contents.

The thermal stability of the samples was also measured using TGA analysis. The results are shown in Figure 5. A mass loss of about 10% was recorded by 200 °C, which can be due to the loss of acidic functional groups in either ASC or GO. While the mass loss was then carried out with the same rate till 30% at 350 °C in samples without GO contents, a higher thermal stability was observed from the higher residual mass of 80%, 76%, 74%, and 71% by 350 °C in F2-16, F1-8, F1-16, and F2-8. Then, the samples immediately encountered a significant decrease in temperature ranges from 350 °C to 443 °C for F1-16, F2-16, and F2-8, this rapid mass reduction occurred for F1-8 in a wider range of temperatures between 350 °C to 460 °C. Additionally, F1-16 exhibited a higher residual mass of 23% at 550 °C in contrast to other samples with GO contents; 19%, 11%, and 7% for F1-8, F2-16, and F1-8, respectively. These results suggest that GO contents and branching densities are both responsible for thermal stability of nanocomposite, since a more stability was observed in higher GO contents and branching densities (F2-16).

**Figure 6:**
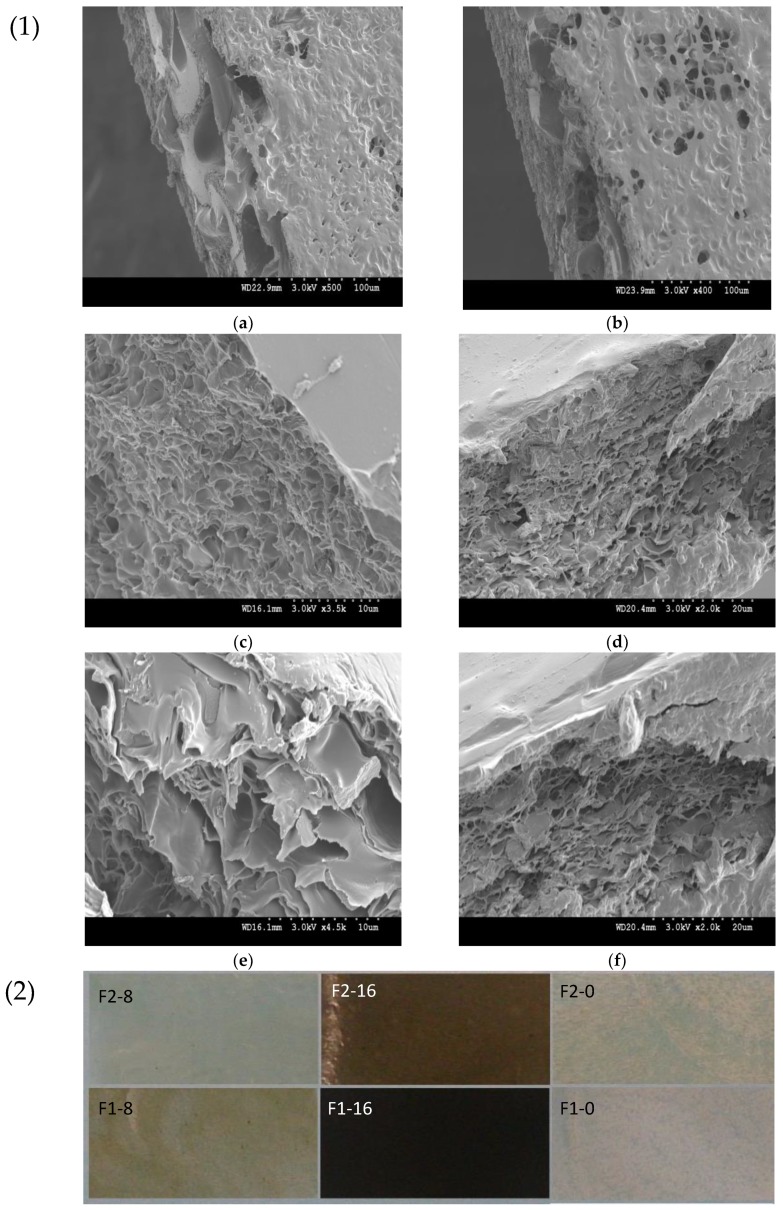
(**1**) Investigation of cross-sectional morphology of porous composite films of ASC-g-P(MMA-co-EA) without GO contents: (**a**) F1-0, (**b**) F2-0; the multilayer nanostructure of ASC-g-P(MMA-co-EA) with GO contents: (**c**) F1-8, (**d**) F1-16, (**e**) F2-8, (**f**) F2-16; and, (**2**) the color appearances of nanocomposite films on a light blue substrate.

To investigate further, the morphology of the film samples was analyzed via SEM (Figure 6). SEM images of samples without GO contents clearly show a porous structure that was caused by amphiphilic nature of ASC-g-p(MMA-co-EA) in casting method, forming a self-assembly hydrogel system. However, smooth surface and a layered inner structure were observed in SEM images of samples with GO contents. This is due to the presence of graphene oxide layers in nanoscale providing higher possibilities for both polar and nonpolar segments of GO-ASC-g-P(MMA-co-EA) complex to conjugate during the morphological transformation from fluid to film. Additionally, from the SEM images of the samples with GO contents, not clearly GO agglomeration was observed, confirming the high exfoliation and uniform dispersion during in-situ polymerization and spatially multilayer film formation, afterward.

Furthermore, appropriate mechanical responses play a key role for any matrix-filler to be considered for a range of applications. Hence, the mechanical properties of the film samples were investigated through an Instron tester. For each sample, five specimens were prepared. Each specimen was held between the jaws of the Instron Tensile Tester applying load until breaking. The samples without GO contents were studied as the reference to investigate the effect of GO loadings on GO-ASC-g-P(MMA-co-EA). Modulus and tenacity were calculated for each specimen by software by dividing the load (N) to the area (mm^2^). The mean and standard deviation values of mechanical properties were calculated for each group of specimens (*n* = 5), Table 2.

**Figure 7:**
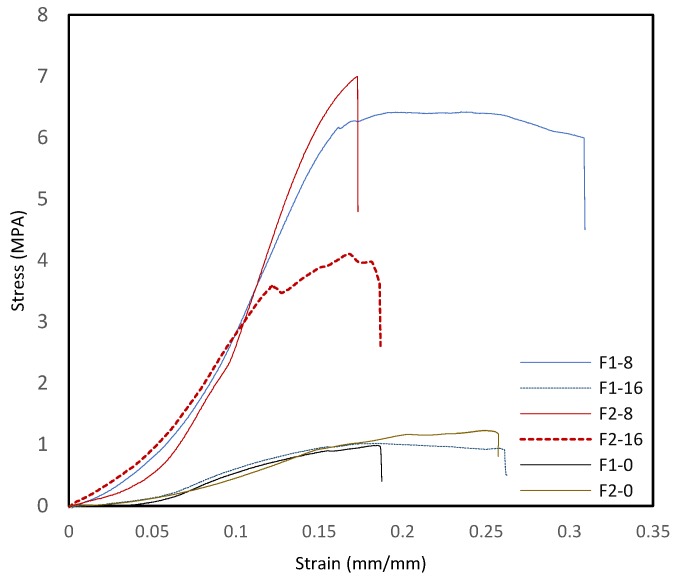
A representative stress-strain curve recorded from tensile test of composite films from ASC-g-P(MMA-co-EA) with/without GO contents.

Modulus and tenacity refer to the stiffness and strength of the samples, respectively. This measurement was performed at the ratio of tenacity to strain, wherein the tenacity was calculated based on the breaking force divided by the initial mass of the samples, representing the mass stress at break. Representative stress-strain curves of GO-ASC-g-P(MMA-co-EA) are shown in Figure 7. All the samples exhibited elastoplastic behavior. The mean values of Young’s modulus (*E*), the tenacity, the tensile stress (δ), the tensile strain(ε), and the deformation at break of the samples were reported in Table 2. The variation of the mechanical properties as a function of composition clearly shows a significant enhancement in the studied samples with GO contents as compared to those samples with no GO content.

The results indicate that GO is effective in reinforcing the films that can be appointed as a result of two main reasons; one can be the role of GO during in situ polymerization referring to enhanced grafting performance and grafting efficiency, wherein higher chain entanglement can be achieved with increased branched density along with inter and intramolecular interaction of the components and the other reason can be the multilayer morphology of the films in nanoscale when the nano-layers and intermolecular conjugations conflicting with deformation mechanism. Additionally, from higher strain at break in F1-8 and F1-16, it seems larger elongation can arise in samples with less branching density, while the branching density can provide higher chain entanglement in the elastic phase (F2-8 and F2-16). More specifically, in samples with GO content, the enhancement in tensile strength was observed for F1-8 and F2-8 with less GO contents, whereas the greater GO content did not cause further improvement of the mechanical properties confirming the challenges that should be considered over GO loadings into composite components as a cause of inhomogeneity, as mentioned in thermal studies that can reduce the molecular dynamics. 

**Figure 8:**
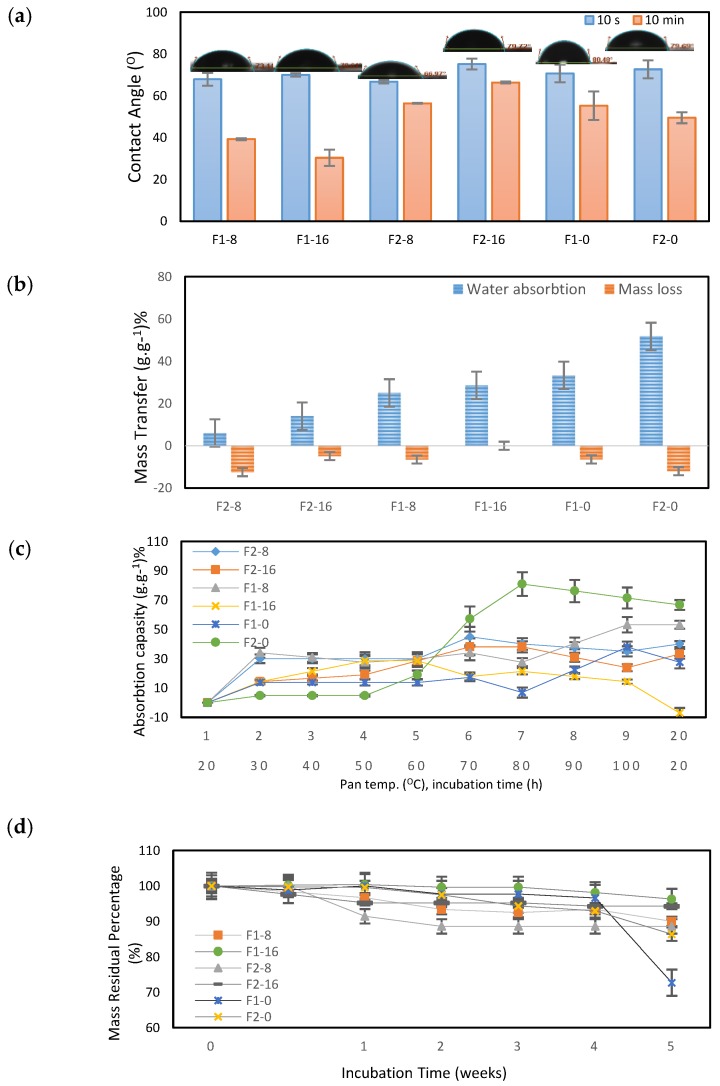
(**a**) The water contact angle vs. time after 10 s and 10 min; (**b**) water absorption (hydration degree) and mass loss after incubation time of 12 h at room temperature; (**c**) Water absorption capacity of the samples as a function of temperature in percentage; and, (**d**) Mass residual percentage vs. incubation time (week).

To better understand the influence of the presence of GO on ASC-g-P(MMA-co-EA), surface wettability, water absorption, and degradation properties of the samples were studied. The results of contact angle measurements are shown in Figure 8a. The average contact angles (CA) of the samples in first 10 seconds are about the same value (CA < 80°) representing the high hydrophilicity of the film surface, even though after 10 min varied surface wettability was observed. More specifically, the samples with GO contents and lower hydrophobic segment (F1-8 and F1-16) showed higher surface wettability after 10 min, whilst F2-8 and F2-16 possessing higher hydrophobic segment, showed lower surface wettability. This result shows that a hydrophobic segment of the composite can have a higher influence on surface wettability as an inhibitor by contrast to GO loadings. Additionally, water absorption of the samples was studied in 12 h at room temperature and then dried in a vacuum oven until a constant weight was achieved. As shown in Figure 8c, mass transfer rate decreased initially in samples with the increased hydrophobic segment of P(MMA-co-EA) in the nanocomposite, whilst increasing the GO content can influence water absorption degree in 12 h as a secondary factor. Meanwhile, a portion of the collagen-based material is expected to be dissolved during the one set soaking in water. This denaturation mostly occurred due to the severe solvents to process the bulk collagen-based copolymer. Even though we used a mild mixed solvent, a small fraction of mass loss was observed in the samples with the exemption F1-16 with the lower hydrophobic segment and higher GO content. Interestingly, in the samples with GO content, a smaller mass loss was observed with increasing GO loadings. This can be due to additional bonds that can be happened between GO and ASC segment of nanocomposite during solubilization and/or solidification. This result indicates that the presence of GO can act as an inhibitor factor to ASC segment to be denatured by the effect of solvents.

To further investigate the water absorption capacity of the samples, as shown in Figure 8c, the water intake of the samples was measured as a function of temperature. Hence, the samples were heated up to 100 °C and then cooled overnight. 

From room temperature to 60 °C, the samples showed almost a slow water intake rate of 10–30%. In temperatures between 60 °C to 100 °C, the water uptake ratio was increased between about 10–50%, with the exemption of F2-0 which was far away from the other samples. In the range of 10–50%, the highest water uptake was for F1-8 and the lowest ratio was for F1-16. This two samples with the lower hydrophobic segment had almost the same water uptake at 50 °C, whilst F1-8 showed upward water uptake ratio and F1-16 reacted in an opposite direction.

A shrinkage was observed in F2-8 and F2-18 in temperatures above 80 °C and then showed an increased water uptake after the cooling phase. This response can be due to reaching the glass transition temperature of the hydrophobic segment that caused obtaining more chain flexibility to agglomerate by the increasing temperature and probably released the hydrophilic segment to absorb water during the cooling phase. In degradation study, as shown in Figure 8d, apart from the sample behaviors during the incubation time, the samples with GO contents showed almost a small gradient of mass loss in contrast to the samples without GO contents that experienced a significant degradation in the fifth week. Interestingly, F1-16 with high GO contents and low density of grafted copolymer showed the highest resistance to mass loss and F2-16 displayed the second stability. This is in agreement with mechanical parameters that cause a decrease in load bearing capabilities, which is probably due to a reduction in molecular dynamics. These results show that within a steady temperature, e.g. room temperature, the presence of GO can deter the degradation rate of ASC-g-P(MMA-co-EA) significantly in contrast with high monomer feed ratios. This can be due to the existence of supplementary bindings, such as hydrogen bonding between GO and collagen graft copolymer that can be stronger than those of between water molecules and the collagen graft copolymers [38]. During all measurements in contact with water, the samples retained their original form, and no migration of GO into the water was observed.

Even though it is less problematic to process amorphous polymeric nanocomposites into molds with a variety of shapes and dimensions to create complex three-dimensional (3D) objects [39,40,41], the fiber formation can represent new challenges to amorphous macromolecular polymeric systems when it comes to Raleigh instability of branched structure of ASC-g-P(MMA-co-EA) and two-dimensional (2D) geometry of graphene nano-sheets in which the dimensions of GO nano-sheets are comparable to fiber diameters. We used the electrospinning technique that allows the alignment of composite fibers in low solution concentration. 

**Figure 9:**
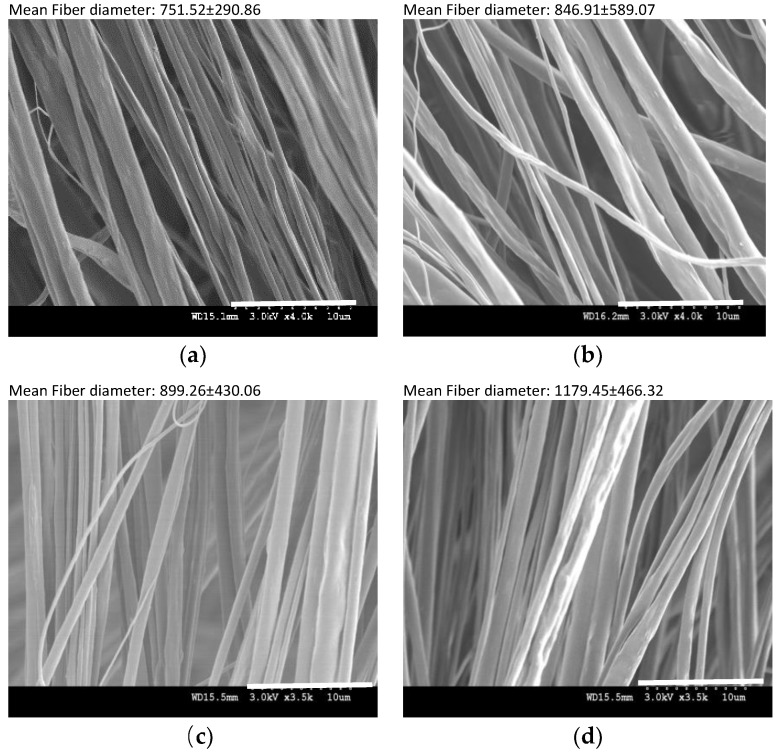
The effect of the GO contents on the mean fiber diameter and the uniformity (standard deviation value) of the electrospun fiber from the GO-ASC -g-P(MMA-co-EA nanocomposite: SEM images of (**a**) M1-8; (**b**)M1-16; (**c**) M2-8; and, (**d**) M2-16; the scale bar shown on the bottom bar is 10 µm.

This was due to deteriorating the destructive impact of Raleigh instability of branched structure of ASC-g-P(MMA-co-EA) in fiber formation. Hence, to study the effect of the GO contents in fiber formation, a set of four solutions of GO-ASC-g-P(MMA-co-EA) with GO contents were successfully electrospun, Figure 9. Electrospinning conditions that used to fabricate the fibers (environmental and experimental parameters) were optimized to obtain fibers along the fiber axes. Figure 9 shows the fiber morphologies representing the mean fiber diameter and the corresponding standard deviations that were calculated to identify the uniformity of the nanofibers in diameter.

Even though the presence of GO in the composite fibers does not seem to affect the alignment morphology and/or bead formation, the uniformity of the nanofibers was affected by both GO and branch density, since the less fiber diameter (751.52 nm) as well as higher smoothness and uniformity (290.86 nm) was observed in F1-8 possessing lower GO contents and branch density.

The decrease in the electrospun fiber diameter with GO contents has been reported in various polymeric systems and attributed to the increased conductivity of the electrospinning solution with GO addition, resulting in a small fiber diameter [42,43,44]. The increased conductivity has been also claimed as the GO is functionalized by amino groups [5,11,45]. By contrast, GO nano-sheet can appear as an electrical insulator, due to the disorder of its sp2 bonding networks as a product of strong acid/base treatment [46,47]. Therefore, the results of fiber morphology are likely attributable to more complex combination of causes; e.g. the presence of GO with comparable size to the fiber diameter when it comes along with branched structure of ASC-g-P(MMA-co-EA) that can intensely influence the dielectric constant and viscosity of the solutions and vary the response to the electric fields eventually.

**Figure 10:**
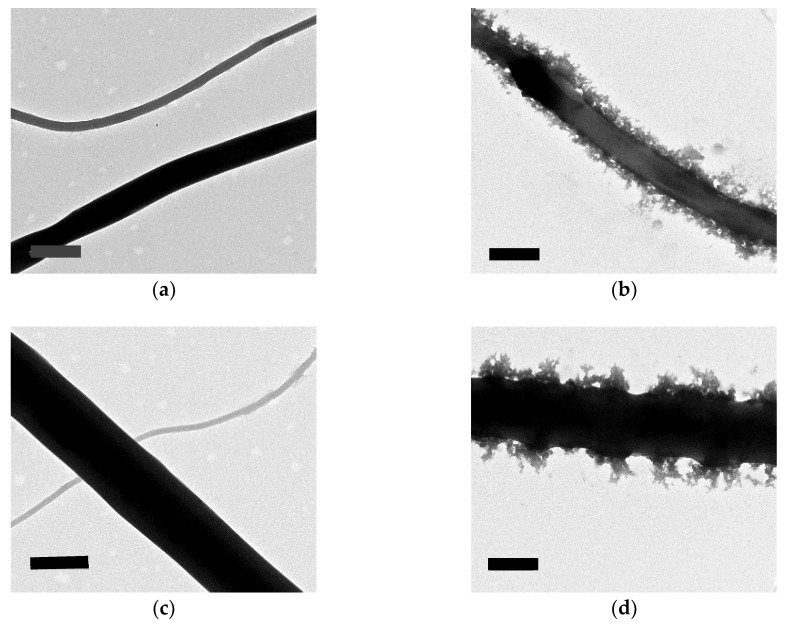
The arrangement of GO in the electrospun fiber from the GO-ASC-g-P(MMA-co-EA nanocomposite: TEM images of (**a**) M1-8, (**b**) M1-16, (**c**) M2-8; and, (**d**) M2-16. The scale bar shown on the bottom bar is 500 nm.

Finally, to better understand the GO arrangement in the structure of the fibers we studied the TEM image of the electrospun fibers of the samples with GO contents. As shown in Figure 10, distinguished appearance can be observed on the surface of the nanofibers bearing more GO contents (M1-16, and M2-16) as compared with nanofibers with less GO contents (M1-8 and M2-8). Even though this notable GO decoration on fiber surface can be beneficial for applications that require highly active functional groups of GO on the surface of the fibers, the addition of GO contents can simply increase the average fiber diameter and decrease its uniformity. 

## 4. Conclusions

The reaction of a free radical polymerization onto acid soluble collagen with and without the presence of GO was proposed to investigate the grafting performance and the grafting efficiency. The grafting performance of the collagen graft copolymer was enhanced by increased comonomer feed ratios from 16.09 wt.% to 46.48 wt.% at about 33.70 ± 3.03% of the efficiency of methyl methacrylate-co-Ethyl Acrylate. Under the same reaction conditions, the grafting performance and efficiency were further enhanced up to 68.7 wt.% and 97.63% with the incorporation of 1.8 wt.% of GO. This suggested that the presence of GO accelerated the polymerization reaction and that it also acted as a coupling reagent to bond ASC and homopolymerized P(MMA-co-EA) within ASC-g-P(MMA-co-EA) copolymer. The tensile strength of the GO-ASC-g-P(MMA-co-EA) copolymer was enhanced as compared to the corresponding copolymers without the presence of GO. By the addition of 3.6 wt.% of GO, the improvement in grafting performance was only achieved while the grafting efficiency did not achieve a significant increase. The processability of the obtained nanocomposites was investigated through a casting method and nanofiber electrospinning. Due to the geometric 2d shape of GO nano-sheet coming along with the branched structure of ASC-g-P(MMA-co-EA), the resulted network is easier to be processed through a casting method. A multilayered composite structure of the nanocomposite films is an attractive candidate for novel applications in nanomechanical systems and paper-like collagen-based composites. The coupling interaction of the GO contents also reduced the degree of swelling of the nanocomposite films and decelerated the degradation rate. The smoothness and the uniformity of the nanofibers can be affected by the GO in high loadings, whereas the lowest fiber diameter can be achieved in less branching densities and low GO contents.

## Figures and Tables

**Table 1:** The effect of graphene oxide (GO) contents on grafting parameters.

Sample	Comonomer Cont. in Feed (mmol)	EA Cont. (%)	Initiator (mmol)	GO (13 mg mL^−1^) Content (mL)	*GP* (%)	*GE* (%)	Nitrogen Content (%)
S1	54.91	0.5	4.51	0	16.09	30.67	7.46
				8.00	68.7	97.63	6.32
				16.00	94.47	97.42	6.03
S2	109.85	5.00	9.10	0	46.48	36.74	6.86
				8.00	101.66	94.02	5.65
				16.00	108.93	87.90	5.96

**Table 2:** Mechanical properties of samples.

Samples	Tensile Stress (N/mm^2^)	Tensile Strain (mm/mm)	Tenacity (N/mm^2^)	Breaking Stress (N/mm^2^)	Breaking Strain (mm/mm)	Modules (CN/mm^2^)
F1-8	6.42 ± 0.96	0.20 ± 0.15	15.11 ± 2.57	6.00 ± 0.90	0.31 ± 0.05	84.52 ± 13.13
F1-16	1.02 ± 0.20	0.19 ± 0.04	2.72 ± 0.54	0.86 ± 0.17	0.26 ± 0.06	14.66 ± 2.93
F2-8	7.00 ± 1.40	0.17 ± 0.09	16.66 ± 1.87	6.99 ± 0.70	0.17 ± 0.16	107.85 ± 9.79
F2-16	4.11 ± 0.49	0.17 ± 0.02	9.95 ± 1.31	3.66 ± 0.44	0.19 ± 0.04	61.41 ± 7.73
F1-0	0.99 ± 0.09	0.18 ± 0.08	2.63 ± 0.24	0.71 ± 0.06	0.19 ± 0.02	14.31 ± 1.29
F2-0	1.23 ± 0.07	0.25 ± 0.05	3.28 ± 0.20	1.13 ± 0.07	0.26 ± 0.03	13.37 ± 0.80
